# Right hemisphere engagement in language abilities in older adults: indication of compensation rather than decline

**DOI:** 10.3389/fragi.2025.1458692

**Published:** 2025-06-02

**Authors:** Daniel Solomons, Maria Rodriguez-Fernandez, Francisco Mery-Muñoz, Leonardo Arraño-Carrasco, David Toloza-Ramirez, Francisco Sahli-Costabal, Carolina Mendez-Orellana

**Affiliations:** ^1^ Institute for Biological and Medical Engineering, Pontificia Universidad Católica de Chile, Santiago, Chile; ^2^ Milenium Institute for Intelligent Healthcare Engineering, Santiago, Chile; ^3^ Department of Neurosurgery, Faculty of Medicine, Pontificia Universidad Católica de Chile, Santiago, Chile; ^4^ Department of Radiology, Faculty of Medicine, Pontificia Universidad Católica de Chile, Santiago, Chile; ^5^ School of Speech Therapy, Faculty of Rehabilitation Sciences, Exercise and Rehabilitation Sciences Institute, Universidad Andres Bello, Santiago, Chile; ^6^ Interdisciplinary Center for Neuroscience, Faculty of Medicine, Pontificia Universidad Católica de Chile, Santiago, Chile; ^7^ Department of Mechanical and Metallurgical Engineering, School of Engineering, Pontificia Universidad Católica de Chile, Santiago, Chile; ^8^ Speech and Language Pathology Department, School of Health Sciences, Faculty of Medicine, Pontificia Universidad Católica de Chile, Santiago, Chile

**Keywords:** aging, gray matter volume, language lateralization, magnetic resonance imaging, phonological, semantic, syntactic, verbal fluency

## Abstract

**Introduction:**

Structural brain changes during aging have been used as specific markers to distinguish normal aging from dementia. Changes in specific cognitive abilities such as episodic memory, processing speed, and executive functions, are observed in healthy aging. Limited evidence reports changes in linguistic functions alongside structural and functional brain changes. This study investigates correlations between language performance, gray matter volume (GMV), and neural activity in language regions, adjusted for demographic factors, in healthy older adults.

**Methods:**

Twenty-seven right-handed participants aged 60–87 were evaluated for overall linguistic performance using the Spanish version of ScreeLing (SCL) test and phonemic fluency and semantic verbal fluency tasks (PF and SF). Participants also underwent an MRI session during which they performed a functional MRI language task. T1-weighted MRI scans were used to measure GMV in specific language-related regions and assess language lateralization. Correlational analyses were conducted between language scores, GMV, years of education, age, sex, and fMRI lateralization.

**Results:**

In the right hemisphere (RH), significant positive correlations were found between SCL scores and GMV in the orbital inferior frontal gyrus (r = 0.5402; p = 0.0044) and the superior temporal gyrus (r = 0.516; p = 0.007). Furthermore, SCL and Phonemic fluency positively correlated with years of education, indicating that higher education enhances speech performance. No significant correlations were found in the left hemisphere (LH). Age, sex, and fMRI lateralization did not significantly correlate with specific linguistic scores.

**Discussion:**

These results challenge the current view of the role of the right hemisphere in language performance as increased GMV in specific right hemisphere language regions is associated with better language performance, highlighting the role of the right hemisphere in supporting language skills during healthy aging.

## Introduction

As aging is associated with significant deterioration of the brain, age-related neural changes underpin alterations in cognitive functions (Salthouse et al., 2009). Neuroimaging studies aim to differentiate between normal and pathological aging, particularly focusing on language function as an early marker (Gorno-Tempini et al., 2008). It is crucial to note that structural analysis alone often fails to predict the early stages of dementia, highlighting the necessity for comprehensive assessments that include functional and behavioral markers such as language proficiency to better understand the trajectories of cognitive decline in aging populations.

The study of gray matter, where neuronal cell bodies are contained, is essential for understanding neural processing in the brain (Kandel et al., 2000). Voxel-based morphometry (VBM), which measures gray matter volume (GMV) from a structural MRI scan, is a popular method employed for studying the link between the brain and various behaviors and biological processes. In the study of normal brain aging, several GM markers have been identified. For example, Lemaitre et al. (2012) and Kwak et al. (2018) have associated aging and personal perceptions of the aging process with GMV changes. These changes are part of a broader spectrum of biological processes linked to GMV alterations, including memory, schizophrenia, and cognitive decline associated with aging (Ashburner and Friston, 2000; Boller et al., 2017; Chatterjee et al., 2020; Laubach et al., 2018). However, the specific patterns of GMV changes during normal aging and their implications for language processing remain the subjects of ongoing research.

Language performance has been established as an early marker to detect pathological aging (Ahmed et al., 2013; Gagliardi, 2024; Olmos-Villaseñor et al., 2023). Studies have shown that fMRI signals in regions of the brain responsible for language activity are correlated with GMV, indicating the reliability of GMV for studying language performance (Dorsaint-Pierre et al., 2006; Jansen et al., 2010; Josse et al., 2009; Labudda et al., 2012). GMV in the language regions of the brain has been associated with language performance in older adults, with GMV increases in temporal language areas and other regions being linked to increased language task performance (Roehrich-Gascon et al., 2015; Tremblay and Deschamps, 2016; Zhang et al., 2013). Furthermore, better verbal fluency (VF) performance has been linked to higher GMV in superior parietal clusters (Pelzer et al., 2017). Increased GMV in language regions is also associated with bilingualism, potentially due to the higher cognitive demands in those regions that are a consequence of speaking two languages (Green et al., 2007; Güntürkün et al., 2020; Mechelli et al., 2004).

Advances in neuroimaging techniques have made it possible to identify a broader brain language network involved in speech production and comprehension. Language regions involved in language processing are generally reported as lateralized to the left hemisphere (LH); however, the right hemisphere’s (RH) importance in language has been increasingly highlighted (Binder and Desai, 2011; Hickok and Poeppel, 2007; Price, 2012). The specific importance of the right hemisphere in language processing has been emphasized in numerous studies (Lindell, 2006; Pinel and Dehaene, 2010; Pujol et al., 1999; van Ettinger-Veenstra et al., 2010). Regarding the right hemisphere and GMV, pediatric studies have linked right hemisphere GMV in the language regions to various speech-related disorders (Kurth et al., 2018; Ligges et al., 2022; Pigdon et al., 2019). However, there is limited evidence of a specific correlation between RH GMV and language, apart from studies exploring its possible compensatory role in LH stroke patients (Hope et al., 2017; Lukic et al., 2017; Xing et al., 2016).

The literature suggests a complex interplay between brain function and structure in the context of aging. Although studies have correlated reduced language test scores with both decreased GMV and gray matter damage, alongside diminished neural activity in bilateral temporal and parietal language regions, the exact nature of the relationship between the structural and functional changes in the aging brain remains a topic of ongoing investigation (Amici et al., 2007; de Zubicaray et al., 2011; Montemurro et al., 2023; Richardson and Price, 2009; Stein et al., 2012). Understanding this functional/structural interplay is critical for establishing the mechanisms underlying language processing.

Despite evidence associating increased fMRI activity and GMV in the language areas with higher language performance, various questions remain. A large majority of studies focus on fMRI lateralization as an indicator of language performance, a measure that can be highly variable across participants and experimental paradigms due to factors such as venous signal bias and neurovascular uncoupling (Gorgolewski et al., 2012; Morales, 2021; Otzenberger et al., 2005). Boller et al. (2017) established a link between fMRI activity, GMV, and years of education in older adults, but no significant correlation linking these factors to behavioral language task performance was found, displaying complexity in the brain–behavior relationship when taking a range of factors into account. Overall, there is a significant gap in the literature in studies investigating healthy older adults’ language performance and its link to the right hemisphere brain structure differences. A better understanding of this link could be useful in clinical settings when predicting deficits based on lesion location or in healthy older adults, where bilateral GMV decreases are observed together with reduced language performance (Ramanoël et al., 2018; Richardson and Price, 2009).

Based on all considerations mentioned, the current study investigates the correlation between overall language function, measured by the ScreeLing (SCL) test, verbal fluency (semantic and phonemic verbal fluency tasks), and GMV in the language ROIs of healthy older adults. We also examine the link between language scores and years of education and fMRI lateralization (fMRI LI) during a phonemic fluency association task, as well as age and sex.

## Methods

### Participants

A total of 27 right-handed, healthy older adults participated in this study, with ages ranging from 60 to 87. Demographic information of the participants is displayed in Table 1. The exclusion criteria included a history of language or speech disorders, evidence of cognitive decline (as measured by the MoCA), severe hearing impairments, severe visual perceptual disorders, severe motor disabilities, or recent psychiatric or neurological conditions, and contraindications for MRI. Written informed consent was obtained from all participants. Our study protocol was approved by the scientific medical ethical committee of Pontificia Universidad Católica de Chile (ID 220322003).

**TABLE 1:** Participant’s demographic characteristics and language tests.

	Full sample (n = 27)
Age, years mean, (SD) [Range]	67.9 (7.5) [60.0 – 87.0]
Gender, n (%)	
Female	16 (59.3%)
Male	11 (40.7%)
Education (years)	
MEAN (SD) [Range]	13.9 (3.2) 7.0 − 19.0]
Total Intracraneal Volume (TIV)	1358.5 (148.9) [1091.0 − 1676.2]
fMRI language lateralization	
TYPICAL, n (%)	21 (77.8%)
ATYPICAL, n (%)	6 (22.2%)
Behavioural tests	
ScreeLing mean, (SD) [Range]	70.0 (1.9) [65.0 – 72.0]
Phonemic fluency (FAS), mean, (SD) [Range]	40.5 (15.0) [14.0 – 62.0]
Sematic fluency (Animals), mean, (SD) [Range]	19.7 (5.1) [9.0 – 29.0]

Note: SD = standard deviation.

### Collection of behavioral data

#### ScreeLing

Language performance was assessed by the Spanish version of the ScreeLing (SCL) test (Mora-Castelletto et al., 2020) in all participants before undergoing the MRI scanning session. The SCL is a validated test for exploring language performance in healthy people and patients with brain injury and was selected for its low experimenter bias and highest diagnostic odds ratio (El Hachioui et al., 2017). This test evaluates the expression and comprehension of language among three critical subdomains, namely, semantics, phonology, and syntax skills. Each subdomain is scored based on the performance of the subjects, and a global score is obtained. The total score is 72 points, considering a maximum score of 24 for semantics, phonology, and syntax. The cut-off score is 68 points, with scores below 68 indicating language impairment.

#### Verbal fluency

Phonemic and semantic fluency scores were collected using a standardized test where participants are instructed to generate as many words as possible within 1 min—either beginning with the letter F, A, or S (phonemic fluency) or naming animals (semantic fluency)—to assess verbal fluency and cognitive flexibility. According to the methodology of Tombaugh et al. (1999), two scores were obtained: one combining the F, A, and S word generation task and another for the animal naming task. Table 1 displays the demographic information of the participants.

### MRI acquisition

Participants were scanned using a Philips Ingenia 3-T MR System (https://www.usa.philips.com/). A volumetric T1-weighted structural image (echo time = 302 3.2 ms; repetition time = 8.2 ms) with an adequate slice thickness of 1.0 mm was acquired for anatomical registration. Functional images were also acquired using a gradient echo-planar imaging pulse T2*-weighted sequence with echo time = 30 ms as a language task was completed with a repetition time of 3,500 m (the phonemic fluency association task).

### Obtaining gray matter volume values in the language regions of interest

**FIGURE 1:**
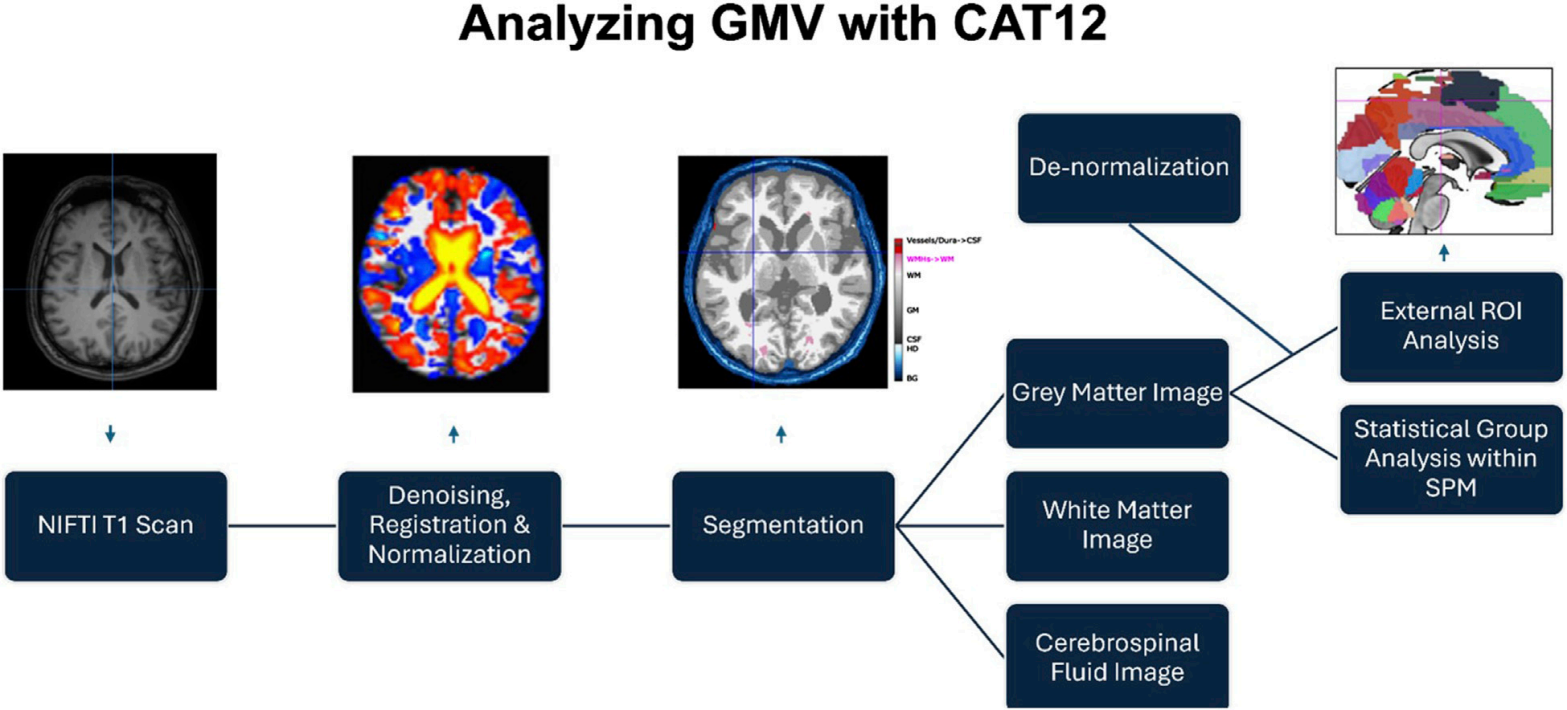
Obtaining the GMV of ROIs in the CAT12 toolbox in SPM12.

With the acquired T1 scan from each individual participant, a GMV measurement was obtained using voxel-based morphometry (VBM) in the CAT12 toolbox in SPM12 (Gaser et al., 2022). Specifically, the GMV of regions of interest (ROIs) implicated in language processing was selected to be examined for its effect on language task performance. A summary of the process is provided in Figure 1. In summary, the VBM procedure starts with the preprocessing of each individual T1 image in CAT12, consisting of denoising, centering, and registering it to a common space (the MNI template), and normalizing it using the DARTEL and Geodesic Shooting template (Ashburner, 2007; Ashburner and Friston, 2000). After normalization, the structural T1 image is segmented into gray matter (GM), white matter (WM), and cerebrospinal fluid (CSF). To extract ROI values personalized to each participant, the normalized GM image is overlaid using a brain atlas (CAT12 uses the “Neuromorphometrics” atlas to define regions of the brain spatially), and this overlaid image is moved back from the MNI-registered and Dartel/Geodesic-normalized space back into the original, native space of the individual brain. This means that a list of GMVs within each ROI is obtained for each participant studied, with values personalized to that participant. To account for each participant’s brain size differences, total intercranial volume (TIV) was present as a control variable in all statistical analyses using the ROI GMV value.

### Selection of ROI values for GMV analysis

Upon parcellation of the brain according to the atlas, seven ROIs were selected to be analyzed for their GMV due to the extensive evidence displaying their importance within the language network (Ardila et al., 2016; Binder, 2015; Levelt, 1989; Seghier, 2013), and they were robustly selected to measure language lateralization (Thakkar et al., 2022). These regions were the angular gyrus, medial temporal gyrus, superior temporal gyrus, supramarginal gyrus, and the triangular, opercular, and orbital sections of the inferior frontal gyrus.

### Functional MRI lateralization measure

FMRI task-based data were also collected to test whether blood oxygenation-dependent (BOLD) activity during a language task within the scanner could also be associated with behavioral language performance or GMV in the language areas. Data from individual subjects were spatially realigned, co-registered to their respective high-resolution structural image, and smoothed. A general linear model was used to generate statistical activation maps to visualize activation related to task performance. Functional MRI activation, in the form of individual t-contrast images during each task, was examined by an expert neuroradiologist who was blinded to the language task conducted and the handedness of the patient. To determine functional language lateralization, the activations were inspected at the same language ROIs as for the GMV ROIs, with the additional supplementary motor area, which was not included in the GMV calculation due to its lack of inclusion in the main anatomical studies examining the language network (Ardila et al., 2016; Binder, 2015; Levelt, 1989; Seghier, 2013) and changes in its GMV being associated with many non-speech related behaviors (Maurer et al., 2018; Pilgramm et al., 2016; Rahayel et al., 2018; Tomassini et al., 2011). Activation was categorized by the neuroradiologist as either left-lateralized, right-lateralized, bilateral, or no activation. The bilateral and right-lateralized categorizations were merged to form an “atypical” category; in contrast, the left-lateralized classification was considered “typical” according to the previous literature and the lower prevalence of right and bilateral-lateralized participants compared to those with left-lateralization (Dym et al., 2011; Keller et al., 2018).

### Statistical analyses

First, Pearson’s correlations were run to test whether language scores were correlated with the demographic factors of age and sex. Since FMRI lateralization is a categorical variable (typical vs. atypical), an ANCOVA was performed to compare SCL total scores between the two groups (with years of education as the covariate; Table 3). The analyses aimed to assess the possibility that any associations between GMV and SCL scores were products of demographic differences or whether fMRI LI influenced SCL scores.

Second, a Pearson correlation was run to test the association between the SCL score and years of education, controlling for age so that the association was focused on years of education rather than on the amount of time available to study.

Third, the seven language ROI GMV values in the right hemisphere, controlling for TIV, were evaluated for their association to the SCL score through Pearson’s correlations, corrected for multiple comparisons with the FDR correction. This was followed by the analysis of the seven language ROI GMV values in the left hemisphere.

## Results

### Relationship between language test scores and age, TIV, and sex

Table 2 displays the results of the correlation analysis between language test scores and their relationship with age and sex. The table displays that there were no significant correlations, displaying that these factors had no impact on language test performance.

**TABLE 2:** Pearson’s r values and level of significance when assessing the correlation between language score’s, age and sex.

		Screeling total score	Phonemic fluency total score	Semantic fluency total score
Age	Pearson’s r	−0.171	0.289	−0.132
	p-value	0.394	0.143	0.512
Sex	Pearson’s r	0.037	0.314	0.055
	p-value	0.856	0.110	0.784

No significant correlations were established between language performance and either sex or age.

### Relationship between SCL test score and fMRI lateralization

Table 3 displays the results of the ANCOVA examining the effect of the fMRI LI score on language test scores while controlling for years of education. The analysis did not yield a significant result.

**TABLE 3:** ANCOVA comparing the effect of fMRI LI on language test scores while controlling for education (years). FMRI LI was not significantly related to language behavioral scores.

Test total Score		
Semantic fluency	F	p
	0.135	0.716
Phonemic fluency	F	p
	1.067	0.312
Screeling	F	p
	3.027	0.095

Note: controlling for “education (years)”.

### Relationship between language test scores and years of education

**FIGURE 2:**
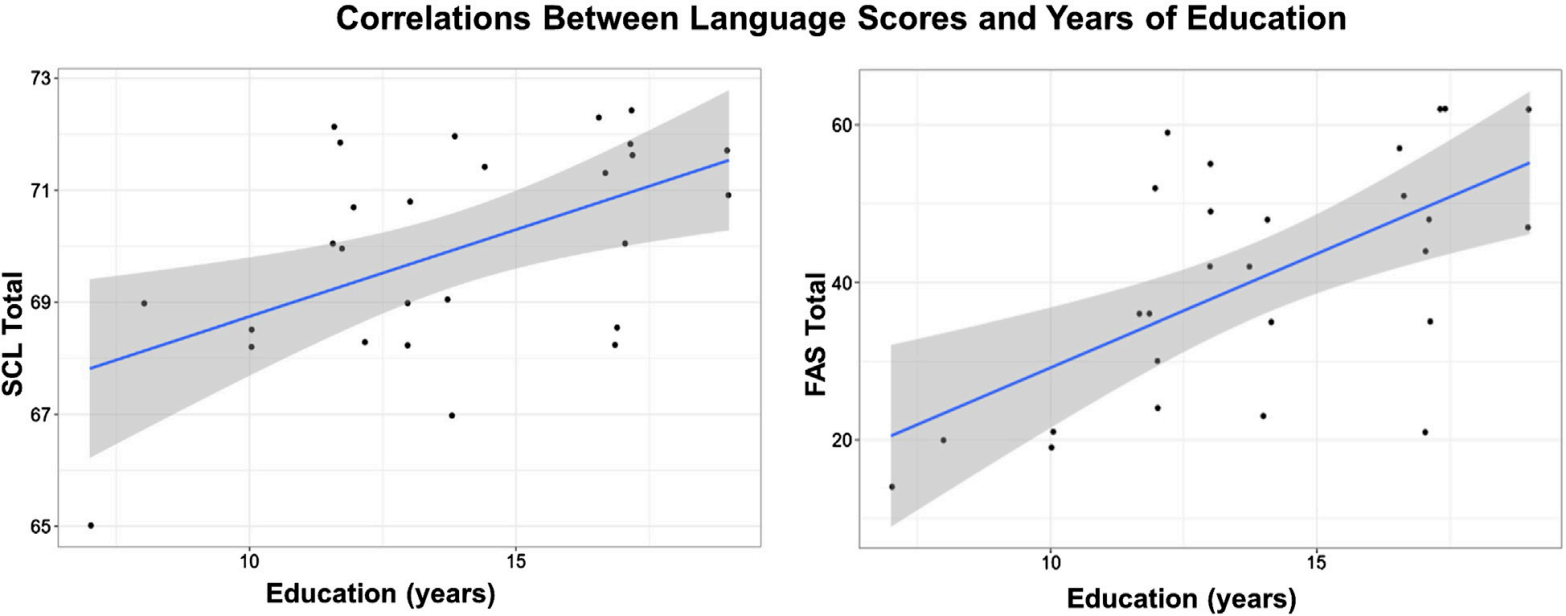
Positive correlation between screeling test score and education (left) and Phonemic fluency and education (right). Controlling for age as covariate.

The years of education of the participants were significantly positively correlated with the total SCL score (r = 0.5356, p = 0.0048) and the phonemic fluency score (r = 0.6324, p = 0.0005), after controlling for age (Table 4; Figure 2). However, semantic fluency score did not display a significant relationship with education. This finding suggests that the amount of education significantly contributes to improved speech performance.

**TABLE 4:** Partial Pearson’s r values and level of significance when assessing the correlation between language scores and education (years), controlling for age as a covariate. Both the FAS and SCL total scores were significantly related to years of education.

Education (years)		Phonemic fluency total score	Semantic fleuncy total score	Screeling total score
	Pearson’s r	0.632***	0.236	0.536**
	p-value	0.001	0.247	0.005

Note: controlling for “age.” Note: *p < .05, **p < .01, and ***p < .001.

### Relationship between GMV and language tests

**FIGURE 3:**
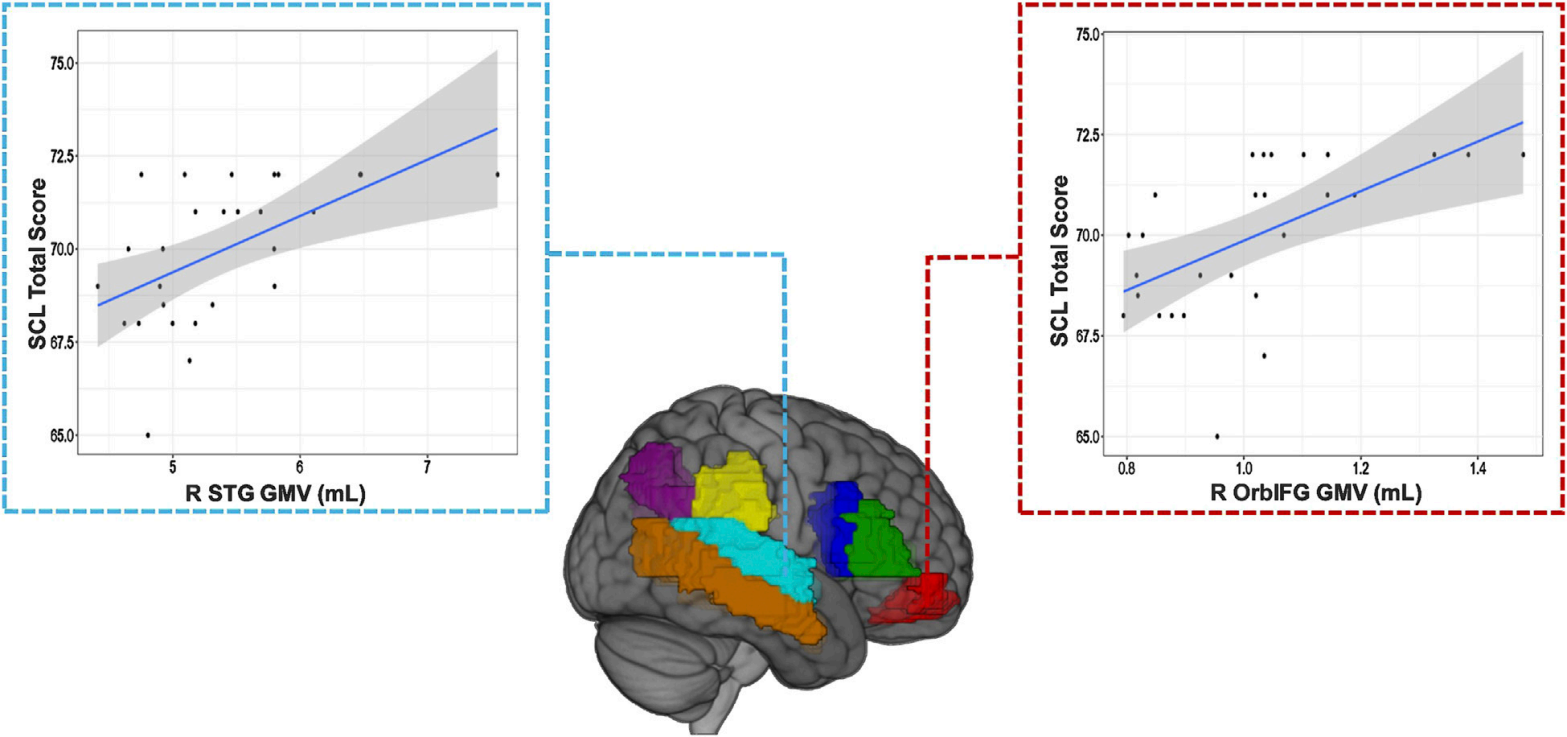
Visualization of the two ROI GMVs that are positively associated with improved SCL language behavioral scores.

The correlational analysis between right hemisphere ROI GMVs and the SCL test score provided two significant positive correlations, both of which remained significant after FDR correction. These were the right orbital inferior frontal gyrus (r = 0.54022; p = 0.0044) and the superior temporal gyrus (r = 0.516; p = 0.007) (Table 5; Figure 3). These findings display that increased right hemisphere GMV in certain regions of the language cortex could aid in improved language performance.

**TABLE 5:** Partial Pearson’s r values and level of significance when assessing the correlation between ScreeLing score and seven right hemisphere language-cortex Regions of interest (ROIs), controlling for Total Intracraneal Volume (ITV) as a covariate.

Right hemisphere ROI GMV (mL)	Screeling total test score		False discovery rate corrected p < 0.05 (✓/✗)
Angular gyrus	Pearson’s r	0.295	
	p-value	0.144	
Middle temporal gyrus	Pearson’s r	0.286	
	p-value	0.157	
Opercular inferior frontal gyrus	Pearson’s r	0.375	
	p-value	0.059	
Orbital inferior frontal gyrus	Pearson’s r	0.540**	✓
	p-value	0.004	
Supplementary motor cortex	Pearson’s r	0.126	
	p-value	0.538	
Supramarginal gyrus	Pearson’s r	0.372	
	p-value	0.062	
Superior temporal gyrus	Pearson’s r	0.516**	✓
	p-value	0.007	
Triangular inferior frontal gyrus	Pearson’s r	0.179	
	p-value	0.383	

Note: controlling for TIV = total intracraneal volume. Note: *p < .05, **p < .01, and ***p < .001.

As the traditionally defined hemisphere for language processing, the left hemisphere ROI GMVs were also studied for their association with language performance. However, Pearson’s correlations, also controlling for TIV, showed no significant relationship between GMV and SCL test scores.

No significance was observed between GMV and phonemic or semantic fluency scores.

## Discussion

The current study explored the relationship between structural and functional brain measurement with two behavioral tests: the verbal fluency task (phonemic fluency and semantic fluency) and an overall measure of language performance measured using the SCL test. Demographic factors such as gender and education were also considered.

In the analyses of GMV in the right hemisphere, the orbital IFG and STG displayed significant positive correlations with the SCL test score, indicating that a higher GMV in these regions contributes to better speech performance. In the left hemisphere, traditionally associated with language processing, no significant correlations were found. When examining demographic factors that could influence language scores, TIV, age, and sex did not show any significant correlations, suggesting that the significant GMV findings are not simply products of age, sex, or size differences, with TIV, usually an indicator of demographic differences, used as a covariate in GMV statistical analyses to further enforce this. FMRI LI did not have a significant effect on language test scores.

This study shows right hemispheric structural, but not functional, or left hemispheric structural associations between the brain volume and language performance during the language tests. This suggests an important role of right hemispheric brain volume in the language network, alongside the educational background, in supporting language abilities. Furthermore, we consistently found a significant positive correlation between language test scores (two out of the three tests) and years of education, demonstrating that higher education levels lead to significantly higher levels of speech performance.

The findings have several implications in relation to previous work. First, it has been shown that cognitive performance, including language, positively correlates with years of education alongside other factors such as age and socioeconomic status (Folia et al., 2022; Foss et al., 2019; Pliatsikas et al., 2019; Skeide et al., 2016). However, voxel-wise differences were not found in a recent study comparing the GMV of highly and lowly educated older adults (Montemurro et al., 2023). Therefore, the current study’s findings—that language performance improves with education and is associated with GMV in certain language areas, but not with age or sex—are novel, especially given that age is typically linked to cognitive decline (Cocquyt et al., 2023; Salthouse et al., 2009). It suggests that neural and cognitive mechanisms involved in language skills are more plastic and responsive to education than demographic values such as age and sex. This reinforces the importance of stimulating cognitive–linguistic performance through educational interventions, regardless of age or sex. This is especially important considering that despite sex differences in brain imaging are limited, women are at a higher risk of developing speech-related neurodegenerative disorders such as Alzheimer’s disease and experience higher rates of cognitive decline with age—possibly due to educational disparities between men and women (Aggarwal and Mielke, 2023; Folia et al., 2022; Hines, 2020; Wallentin, 2009).

The absence of significance between GMV and phonemic fluency (FAS) or semantic fluency (animal naming) tests underscores the complexity of assessing language-performance relationships in relation to brain structure, although it demonstrates the importance of using multiple tasks to measure language function when looking for meaningful results. While the SCL test revealed significant associations with GMV in specific brain regions, these fluency tests did not exhibit similar correlations, suggesting that not all language assessments capture the same underlying neural mechanisms. Despite the non-significant findings with the fluency tests, the superiority of the SCL test in establishing associations with GMV highlights its effectiveness in probing language–brain relationships. This reinforces the importance of utilizing comprehensive and validated assessments to discern nuanced connections between language abilities and brain structure. Overall, in this study, the SCL test was the most robust measure for uncovering the associations between GMV and language ability.

For comparing GMV volume with SCL scores, our study complements previous work that established a link between GMV and language task scores in healthy older adults. First, in non-language areas, GMV was linked to verbal fluency, indicating a GMV–language performance link, but not in areas specifically considered responsible for language (Pelzer et al., 2017). However, studies have already found links directly to the language network, particularly in the bilateral temporal pole (including parts of the STG and MTG) and bilateral IFG (Roehrich-Gascon et al., 2015; Zhang et al., 2013). The novelty of the current study lies in the fact that while Zhang et al. found correlates in bilateral language regions, our study uniquely found increases in right hemisphere language regions, highlighting the right hemisphere’s compensatory role in language performance. This finding links to studies on stroke patients, where right hemisphere GMV increases in areas such as the STG are linked to better speech performance, suggesting right hemisphere structural plasticity (Hope et al., 2017; Lukic et al., 2017; Xing et al., 2016). Our results suggest that the right hemisphere’s language regions support language function not only in pathological cases but also in healthy older adults. This underscores the brain’s plasticity and suggests that the right hemisphere plays a complementary role in maintaining language abilities in healthy aging populations. In terms of the finding that right orbital IFG GMV was positively correlated with language performance, Gao et al. (2020) found that bilinguals had significantly higher functional connectivity in orbital IFG than monolinguals but with no significant GMV differences. This is contrary with our study, which did not find any fMRI correlates but did find GMV differences. However, it has been shown that studies on bilingual vs monolingual participants provide heterogeneous results that complicate the interpretations (Danylkiv and Krafnick, 2020).

It has been theorized that in the language network, GMV, along with white matter tracts imaged through DTI, is a structural representation of functional organization within the language network (Labudda et al., 2012; Powell et al., 2007). However, this is based on association, and our studies’ finding that structural (GMV)–language performance, but not the functional (fMRI)–language performance relationship, was significant highlights the need to better understand the dynamic functional and structural interactions that underlie language processing (Amici et al., 2007; de Zubicaray et al., 2011; Montemurro et al., 2023; Richardson and Price, 2009; Stein et al., 2012). A possible explanation of a direct dynamic relationship between structural GMV and fMRI signal is that changes in the brain’s structural quality, from the low-cell-body-density neuropil to efficient neural networks underlying language functions, may be dynamically coupled with functional changes in neural activation patterns. Specifically, structural alterations in the right hemisphere may modulate the efficiency of neural processing and connectivity, influencing the brain’s ability to recruit specific regions for language tasks, as reflected in the fMRI signals (Porter et al., 2011).

There are some limitations to this study. First, the sample size of 27 participants is small, which was one of the motives for not conducting any group analysis and instead focusing only on correlational statistics. Second, the ROI approach to VBM may overlook effects in other regions, potentially introducing user bias. However, by correcting for TIV and performing FDR correction on the statistical analysis, we are confident that the GMV results reflect a real effect of both the right STG and right orbital IFG on language performance in the participants included. It should also be considered that correlational analysis cannot establish whether the brain differences are due to less engagement in language activity or whether reduced language activity leads to GMV differences. To establish a casual link, future studies should employ longitudinal designs with monitored language activity engagement over a long-term period, while continuing to measure GMV. This would allow for a baseline measurement of GMV (or other anatomical and functional measures) and language performance to be established, and the degree of language engagement could be controlled for when examining brain changes. Future studies could also build on our findings to assess lateralization differences in younger populations, where structural changes in language areas during adolescence have been observed (Tamnes et al., 2010). Finally, there is potential for deeper analysis of Phonemic fluency by integrating clustering information qualitatively, which could uncover more robust associations in future investigations (Unsworth et al., 2010; Zhao et al., 2013). Future work should also focus on examining language performance across different severity grades of dementia or stages of cognitive decline in order to better understand the potential of language performance as a predictor of abnormal or pathological aging (Ahmed et al., 2013; Gagliardi, 2024).

## Conclusion

Overall, the current study aimed to examine how language performance was impacted by GMV in the language regions and other factors such as fMRI lateralization, years of education, sex, and age in a small cohort of older adults. The findings revealed that the SCL, but not the phonemic fluency (FAS) or semantic fluency (animal naming) task, positively correlated with years of education and GMV in the right hemisphere STG and orbital IFG, two regions increasingly considered important for their compensatory role in pathological conditions, although their links to language compensation in healthy adults are still not well-established. This signifies the potential for targeted educational and clinical neuroimaging strategies to improve speech performance in older adults and establish clear biological markers for speech performance in healthy adults.

## Data Availability

The original contributions presented in the study are included in the article/supplementary material; further inquiries can be directed to the corresponding author.
